# Volumetric evaluation of renal sinus adipose tissue on computed tomography images in bilateral nephrolithiasis patients

**DOI:** 10.1007/s11255-020-02395-0

**Published:** 2020-01-31

**Authors:** Peng Lin, Zeng Min, Gong Wei, Hu Lei, Zeng Feifei, Zha Yunfei

**Affiliations:** 1 Department of Radiology, Renmin Hospital of Wuhan University, Wuhan, 430060 China; 2 Department of Nephrology, The Central Hospital of Wuhan, Wuhan, China

**Keywords:** Renal sinus fat, Computed tomography, Nephrolithiasis, Ectopic fat deposition, Vascular calcification

## Abstract

**Purpose:**

To compare renal sinus fat volume (RSFV) separately within the right and left kidneys between bilateral nephrolithiasis patients and healthy controls.

**Methods:**

This cross-sectional study analyzed patients who underwent unenhanced abdominal computed tomography (CT) divided into nephrolithiasis (*n* = 102) and healthy control (*n* = 130) groups. Age, sex, blood pressure [systolic blood pressure (SBP) and diastolic blood pressure (DBP)], estimated glomerular filtration rate (eGFR), body weight, and height of each participant were extracted. Volumetric renal sinus adipose tissue was measured separately for both kidneys on CT images. Urea, serum creatinine (Scr), uric acid (UA), total serum cholesterol (TCH), serum triglyceride (TG), and serum high- and low-density lipoprotein (HDL and LDL, respectively) cholesterol levels were obtained.

**Results:**

Overall, 232 participants (mean age 47 years, 50% women) were enrolled. There were no differences in sex, DBP, urea, and LDL-cholesterol between the two groups (all *p* > *0*.05). However, nephrolithiasis patients had higher age, BMI, SBP, and RSFV; higher Scr, UA, TCH, and TG serum levels; and lower HDL-cholesterol level and eGFR. Average left RSFV was significantly higher than right RSFV in healthy controls (4.56 ± 2.29 versus 3.34 ± 1.90 cm^3^, *p* < 0.001). A significant relationship between bilateral RSFV, age, BMI, SBP, and eGFR was noted in bilateral nephrolithiasis patients. Multivariate linear regression analysis showed age, BMI, and LDL-cholesterol to be independent predictors of left RSFV, and only BMI was an independent predictor of right RSFV.

**Conclusions:**

Our data showed renal sinus adipose tissue accumulation and the relationship among RSFV, age, BMI, and LDL-cholesterol in bilateral nephrolithiasis patients.

## Introduction

Nephrolithiasis is one of the most common urological conditions with a high recurrence rate. It has been thought to be related to diet and abnormal renal handling of electrolytes over the last several decades. However, recently a large number of epidemiologic studies showed a consistent link between nephrolithiasis and other systemic disorders including cardiovascular disease (CVD), metabolic syndrome (MS), and atherosclerosis (AS) [1–3]. Despite this rationale, the pathogenic mechanism underlying nephrolithiasis is still debated. A recently formulated vascular theory argues that a significant pathological process occurs in the renal interstitium in a pattern similar to that of atherosclerotic plaque formation [4]. Both vascular calcification (VC) and kidney stone can be defined as extraosseous sites of abnormal calcium deposition. Moreover, VC and bone demineralization coexist in nephrolithiasis, suggesting the possibility of common potential pathways leading to increased extraosseous calcium depositions in the kidneys and blood vessels [5].

Increasing evidence suggests that ectopic fat deposition or fat accumulation within and around non-adipose tissues and organs plays an essential role in the development of adjacent anatomical organ dysfunction and systemic disorders [6]. Epicardial adipose tissue (EAT) accumulation is associated with myocardial steatosis, development of atrial fibrillation, and ventricular dysfunction [7–9]. Perivascular adipose tissue (PVAT) accumulation has a pro-inflammatory phenotype, leading to atherosclerosis and hypertension [10–12]. The kidneys are surrounded by the intra-abdominal, retroperitoneal fat depot and tend to accumulate ectopic fat in the renal sinus (RS). RS fat is considerably affected by visceral adipose tissue volume and has characteristics of PVAT [13]. As a new index of ectopic fat depots, RS fat accumulation may also mediate local and systemic effects through excessive mechanical pressure and paracrine effects of locally released adipocytokines or lipotoxicity [14, 15]. It has been previously shown that RS adipose tissue may have an independent association with increased risk of hypertension [16] and chronic kidney disease (CKD) [17, 18] even after accounting for other adiposity measures. Furthermore, a recent study has demonstrated the relationship between RSFV in middle-aged patients suspected to have coronary artery disease and coronary artery calcification [19].

To the best of our knowledge, to date, RS fat has not been evaluated in patients with nephrolithiasis. Thereby, the hypothesis explored in this study was that RS fat accumulation is more prevalent in nephrolithiasis. The present study aimed to determine (1) whether patients with nephrolithiasis have more RSFV than healthy controls and (2) whether RSFV is related to other possible variables in nephrolithiasis.

## Materials and methods

### Participants

The retrospective study sample comprised patients from the Renmin Hospital of Wuhan University, Wuhan, China who underwent unenhanced abdominal computed tomography (CT) between October 2017 and April 2019.

Patients with bilateral punctiform calculi (stones with diameter < 0.5 cm) without obvious clinical symptoms were included. Patients with an underlying cause of urinary calculi, certain systemic metabolic diseases causing abnormal calcium and phosphorus metabolism, hydronephrosis or renal cyst affecting the measurements, congenital anatomical abnormalities of the kidneys, liver disease, chronic intestinal disease, and merging of the tumor were excluded from the analyses. Further, kidney transplant recipients, patients with any autoimmune disease, known coronary artery disease, previous coronary revascularization, chronic diarrhea, or inflammatory bowel disease and patients with a history of stroke were also excluded from the study. According to the inclusion and exclusion criteria, 232 participants (115 men and 117 women; mean age 47 ± 13.6 and 48 ± 12.8 years, respectively) were analyzed. The control group consisted of healthy adults with no kidney stones and signs or symptoms of systematic diseases. All participants provided written informed consent at enrollment. All investigations were performed following institutional guidelines with approval from the institutional ethics review board.

### Protocol development

**Fig. 1:**
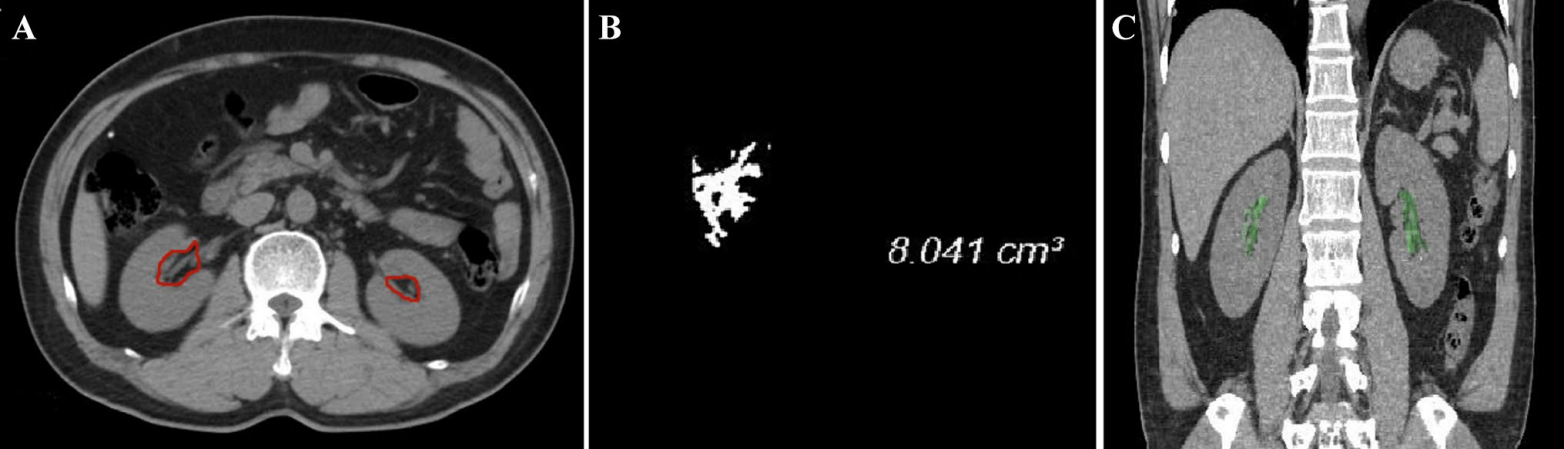
Abdominal computed tomography (CT) scans demonstrating the renal sinus fat measurement technique. **a** Example of a single CT slice selected for renal sinus fat measurement. The reader manually traced within the border of the kidney. **b** One measurement was taken in the left renal sinus. **c** The renal sinus is traced in this figure, highlighting the visualization of fat tissue observed in this region of interest

Unenhanced abdominal CT images were captured using a General Electric medical systems Lightspeed 64-slice CT spiral CT scanner. The CT scanning range covered the abdomen with 0.625-mm slices above the S1 level (tube voltage, 120 kV; tube current, 400 mA; and gantry rotation time, 500 ms). All imaging analyses were performed on a dedicated Advantage Workstation (version 4.7, GE Healthcare). RS fat quantification was performed in a semi-automated method that required a slice-by-slice manual definition of borders on CT axial images, and the total volume of RS fat was obtained. According to Foster et al. [20] protocol, the boundary of RS was defined as a straight line tracing across both dimples at the edge of the RS opening based on visual inspection such that the surrounding abdominal adipose tissue would be excluded from this measurement. Owing to the anatomical differences, a total of 12–18 layers of images were delineated. Pixel density in Hounsfield units (HU) were used to identify RS adipose tissue based on a window width of − 195 to − 45 HU, centered on − 120 HU. RSFV was measured separately within the right and left kidneys (Fig. 1).

Repeated measurements were performed in 22 arbitrarily selected patients by another experienced radiologist to determine the inter-observer variability in RSFV measurements. Inter-observer variability was evaluated using Pearson's correlation coefficient for these measurements on identical images.

## Data collection

Age, sex, body weight, and height of each participant were recorded after their admission. Body mass index (BMI) was calculated as the ratio of weight (kg) to height squared (m^2^). Blood pressure [systolic blood pressure (SBP) and diastolic blood pressure (DBP)] measurements were performed by a professional medical assistant. The estimated glomerular filtration rate (eGFR) was estimated in all subjects using the abbreviated modification of diet in renal disease study equation [21]. Laboratory data included a metabolic panel. Urea, serum creatinine (Scr), uric acid (UA), total serum cholesterol (TCH), serum triglycerides (TG), and serum high- and low-density lipoprotein (HDL and LDL) cholesterol levels were obtained from the clinical examination of a fasting blood sample within 7 days of the CT examination in the Clinical Laboratory of the Renmin Hospital of Wuhan University.

### Statistical analysis

Statistical analyses were performed using the Statistical Package for Social Sciences for Windows version 23.0 (SPSS, Chicago, IL). Dependent variables were analyzed to determine whether they were normally distributed using the Kolmogorov–Smirnov test. Means and standard deviations were calculated for continuous variables. Linear associations between continuous variables were assessed using the Spearman correlation test. Differences between the nephrolithiasis and control groups were tested by Student's *t *test for continuous variables and Chi-square analyses for categorical variables. The right and left RSFVs were compared using paired Student's *t* test in the control group. In the linear regression models, univariate associations between RSFV and other variables (age, BMI, SBP, Scr, UA, TCH, TG, HDL-cholesterol, LDL-cholesterol, and eGFR) were explored. Multivariate linear regressions were performed to determine significant associations between RSFV and other variables (age, BMI, SBP, Scr, UA, TCH, TG, HDL-cholesterol, LDL-cholesterol, and eGFR), adjusted for potential confounders. Bland–Altman plots were utilized to assess potential systematic biases within the repeated measurements. Statistical significance was set at *p* < 0.05.

## Results

### Variables measured between the nephrolithiasis and control groups

**Fig. 2:**
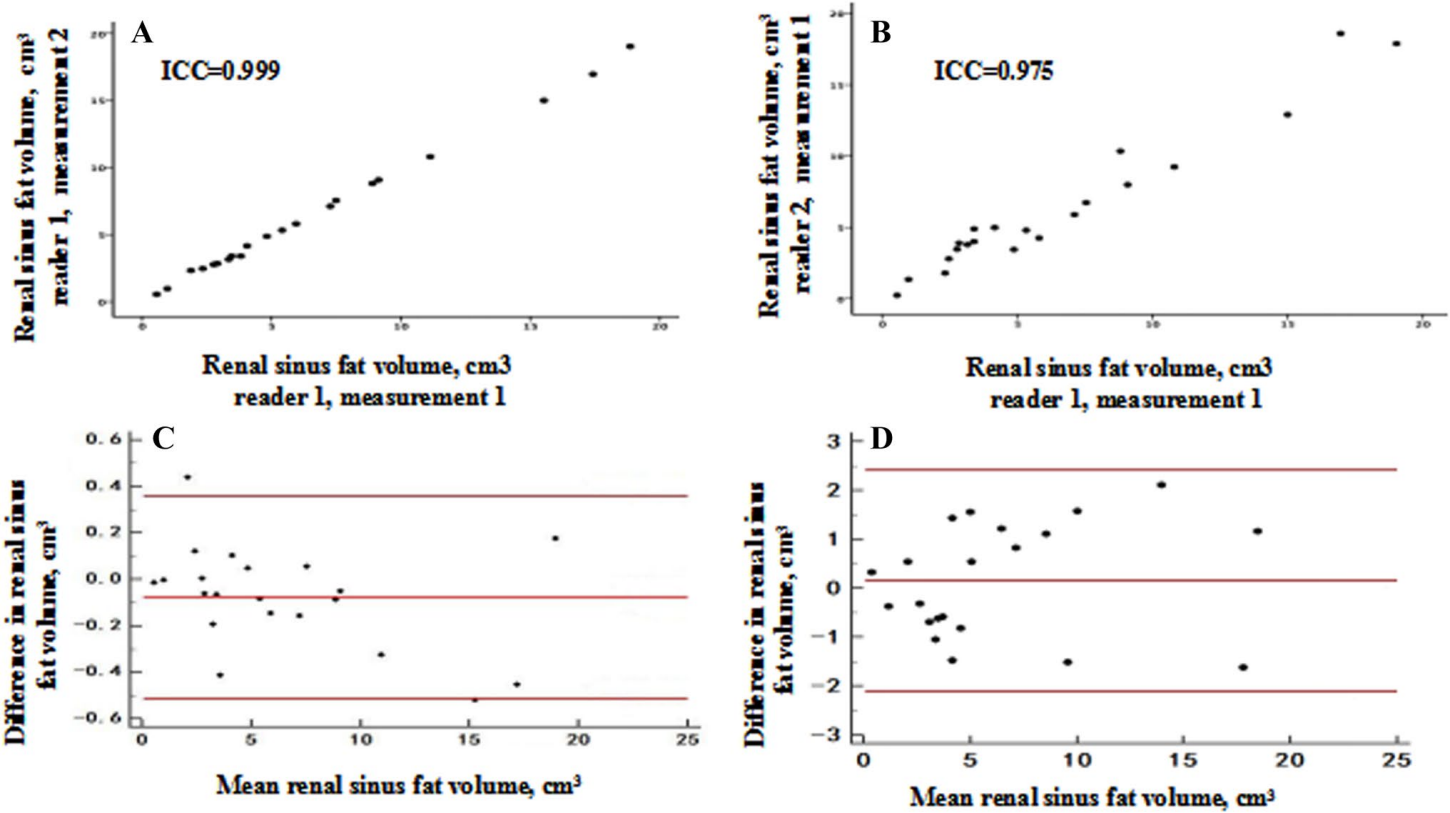
**a**, **b** Renal sinus fat measures between two readers, which are then plotted. Intra-reader inter-class correlation coefficient (ICC) = 0.99 and inter-reader ICC = 0.98. **c**, **d** Bland–Altman plots of the intra-reader and inter-reader renal sinus fat measurements. The average of the repeated measurements is presented on the *X*-axis and the difference between the two measurements is presented on the *Y*-axis. The middle red line represents the mean difference between the repeated measures, and the upper and lower red lines represent the upper and lower confidence limits for the mean difference, respectively

Intra-reader and inter-reader RSFV measurements are plotted in Fig. 2. Pearson’s product-moment correlation coefficient for the RSFV in a sample of 22 patients was *r* = 0.99 for intra-observer variability and *r* = 0.98 for intra-observer variability.

The baseline characteristics of 102 bilateral nephrolithiasis patients and 130 healthy subjects are shown in Table 1. There were no differences in sex, DBP, urea, and LDL-cholesterol between the nephrolithiasis and control groups. However, bilateral nephrolithiasis patients were older and had higher age, BMI, and SBP; higher serum levels of Scr, UA, TCH, and TG; and lower HDL-cholesterol and eGFR than healthy subjects.

**Table 1:** Demographic and biochemical parameters between the nephrolithiasis and control groups

Variables	Nephrolithiasis group (*n* = 102)	Control group (*n* = 130)	*p* value
	Mean (SD)	Mean (SD)	
Age (years)	49.9 (14.0)	45.4 (12.1)	< 0.01
Women (%)	44.12	55.38	0.11
BMI (kg/m^2^)	22.4 (2.0)	20.8 (2.2)	< 0.01
SBP (mmHg)	127.4 (15.7)	118.6 (5.4)	< 0.01
DBP (mmHg)	78.9 (11.6)	77.7 (4.9)	0.26
Urea (mmol/L)	5.38 (1.66)	5.10 (1.24)	0.15
Scr (µmol/L)	74.8 (21.9)	67.8 (16.8)	0.01
µUA (µmol/L)	354.5 (91.0)	316.9 (53.7)	< 0.01
TCH (mg/dL)	4.54 (0.77)	4.97 (0.86)	< 0.01
TG (mg/dL)	1.51 (0.92)	1.25 (0.35)	0.03
HDL-cholesterol (mg/dL)	1.16 (0.37)	1.25 (0.28)	0.04
LDL-cholesterol (mg/dL)	2.63 (0.67)	2.47 (0.60)	0.07
eGFR (ml/min/1.73 m^2^)	93.93 (20.16)	110.11 (10.95)	< 0.01
Left RSFV (cm^3^)	5.47 (3.50)	4.56 (2.29)	0.02
Right RSFV (cm^3^)	4.14 (3.12)	3.34 (1.90)	0.01

All of the participants in the study sample are Chinese

Continuous variables are presented as mean ± SD, and dichotomous variables are presented as %

Data are expressed as mean ± standard deviation (SD). Significance set at *p* < 0.05

*BMI* body mass index, *SBP* systolic blood pressure, *DBP* diastolic blood pressure, *Scr* serum creatinine, *UA* uric acid, *TCH* total cholesterol, *TG* triglycerides, *HDL-cholesterol* serum high density lipoprotein cholesterol, *LDL-cholesterol* low density lipoprotein cholesterol, *eGFR* estimated glomerular filtration ratio, *RSFV* renal sinus fat volume

**Fig. 3:**
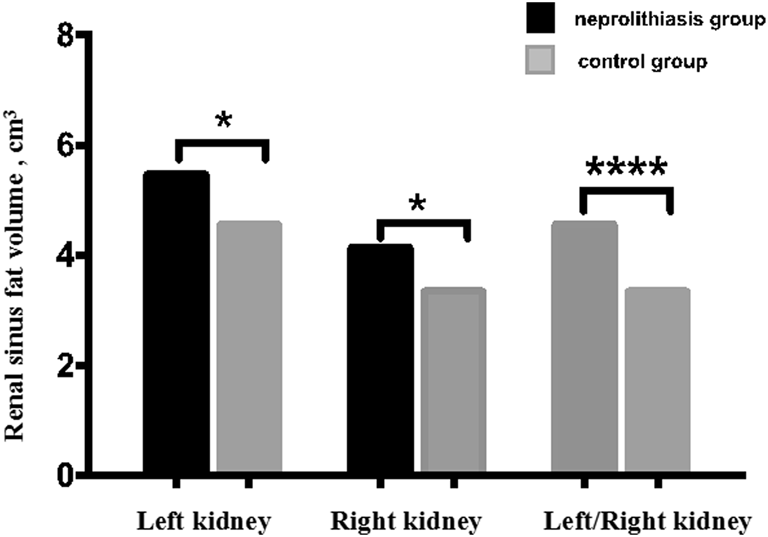
The bilateral (renal sinus fat volume) RSFVs were compared between the nephrolithiasis and control groups, and the left RSFV was compared to the right RSFV in the control group

Bilateral RSFVs were significantly higher in bilateral nephrolithiasis patients than in healthy subjects (left RSFV: 5.47 ± 3.50 cm^3^ versus 4.56 ± 2.29 cm^3^, *p* = 0.02; right RSFV: 4.14 ± 3.12 cm^3^ versus 3.34 ± 1.90 cm^3^, *p* = 0.01). Moreover, the average left RSFV was significantly higher than the right RSFV in the control group (4.56 ± 2.29 cm^3^ versus 3.34 ± 1.90 cm^3^, *p* < 0.001) (Fig. 3).

### Correlation analysis between bilateral RSFVs and other variables in the nephrolithiasis group

There was a statistically significant relationship between bilateral RFSVs, age, BMI, SBP, and eGFR in the nephrolithiasis group (left RSFV: *r* = 0.36, *p* < 0.01; *r* = 0.43, *p* < 0.01; *r* = 0.28, *p* < 0.01; *r* =  − 0.23, *p* = 0.02 and right RSFV: *r* = 0.33, *p* < 0.01; *r* = 0.44, *p* < 0.01; *r* = 0.28, *p* < 0.01; *r* =  − 0.21, *p* = 0.03, respectively) (Table 2).

**Table 2:** Associations of the left and right RSFVs in the nephrolithiasis group with other indices in the univariate regression analysis

Variables	Left RSFV		Right RSFV	
	*r*	*p* value	*r*	*p* value
Age	0.36**	< 0.01	0.33**	< 0.01
BMI	0.43**	< 0.01	0.44**	< 0.01
SBP	0.28**	< 0.01	0.28**	< 0.01
Scr	0.10	0.34	0.06	0.52
UA	0.09	0.37	0.10	0.33
TCH	0.02	0.81	0.06	0.56
TG	0.01	0.97	0.03	0.80
HDL-cholesterol	0.05	0.60	0.05	0.60
LDL-cholesterol	0.17	0.08	0.14	0.17
eGFR	− 0.23*	0.02	− 0.21*	0.03

*BMI* body mass index, *SBP* systolic blood pressure, *Scr* serum creatinine, *UA* uric acid, *TCH* total cholesterol, *TG* triglycerides, *HDL-cholesterol* serum high density lipoprotein cholesterol, *LDL-cholesterol* low density lipoprotein cholesterol, *eGFR* estimated glomerular filtration ratio, *RSFV* renal sinus fat volume

**p < 0.01; *p < 0.05

**Fig. 4:**
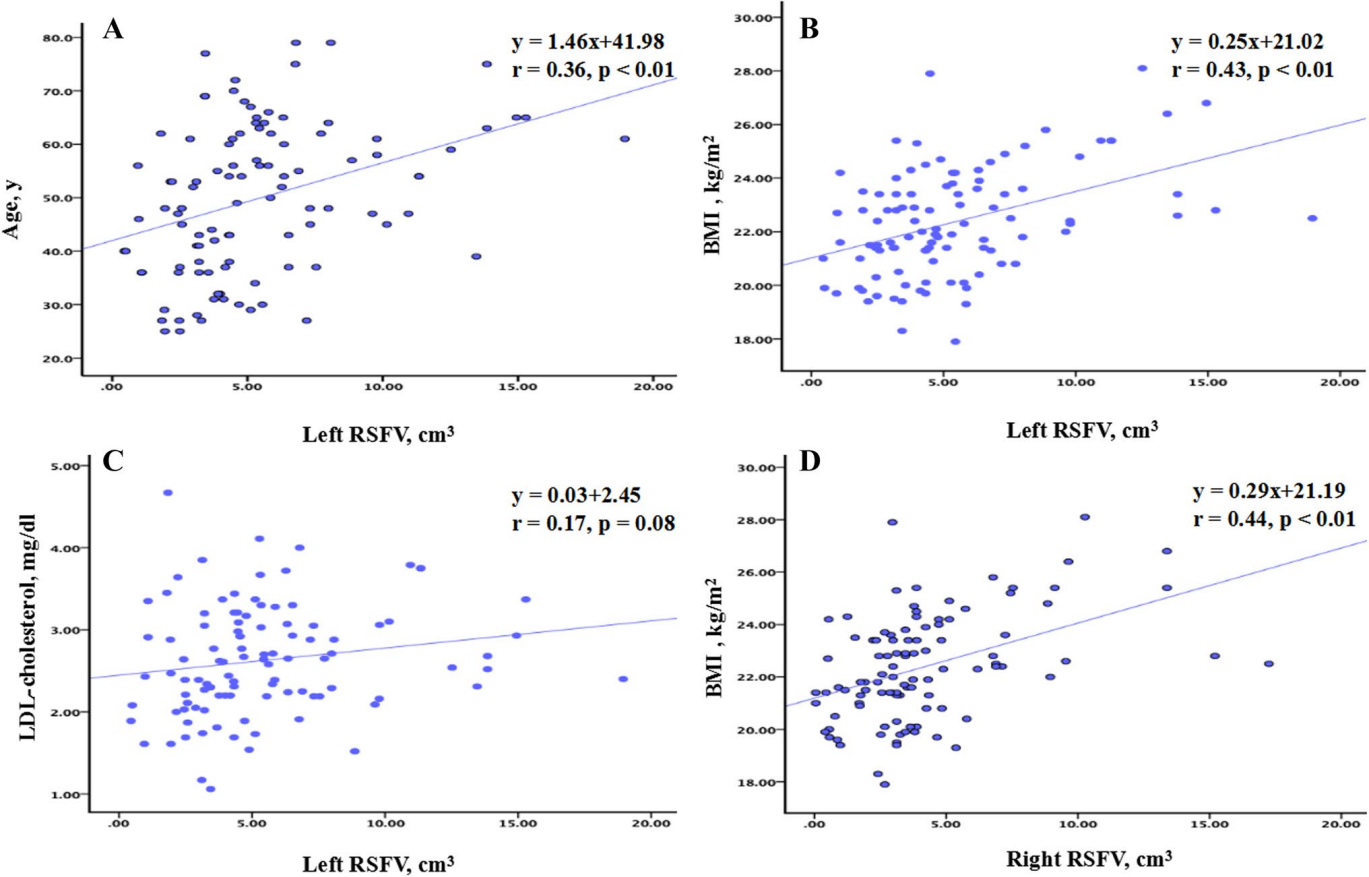
Correlation analysis in the nephrolithiasis group. **a** Between the left RSFV and age. **b** Between the left RSFV and BMI. **c** Left RSFV and LDL-cholesterol. **d** Right RSFV and BMI

The multivariate linear regression analysis revealed that age, BMI, and LDL-cholesterol were independent predictors of left RSFV, and only BMI was an independent predictor of right RSFV. The regression analysis results are shown in Table 3 and Fig. 4.

**Table 3:** Multivariate linear regression analysis with bilateral RSFVs as the dependent variable in the nephrolithiasis group

Variables	Coefficient		SE		*β* (beta)		*p* value		95% CI			
	L-RSFV	R-RSFV	L-RSFV	R-RSFV	L-RSFV	R-RSFV	L-RSFV	R-RSFV	L-RSFV		R-RSFV	
Age	0.07	0.04	0.03	0.03	0.28*	0.17	0.04	0.21	0.01	0.14	− 0.02	0.10
BMI	0.73	0.65	0.16	0.15	0.42**	0.42**	< 0.01	< 0.01	0.40	1.05	0.35	0.95
SBP	− 0.01	0.01	0.02	0.02	− 0.02	0.02	0.87	0.88	− 0.06	0.07	− 0.04	0.04
Scr	0.01	− 0.02	0.03	0.03	0.04	− 0.14	0.86	0.51	− 0.08	0.04	− 0.08	0.04
UA	0.01	0.01	0.01	0.01	0.08	0.13	0.46	0.25	− 0.01	0.02	− 0.01	0.01
TCH	− 0.69	− 0.36	0.51	0.46	− 0.15	− 0.09	0.18	0.44	− 1.70	0.31	− 1.27	0.56
TG	− 0.15	− 0.11	0.35	0.32	− 0.04	− 0.03	0.67	0.73	− 0.86	0.55	− 0.75	0.53
HDL-cholesterol	0.69	0.40	0.93	0.84	0.07	0.05	0.46	0.64	− 1.16	2.53	− 1.27	2.08
LDL-cholesterol	1.13	0.73	0.53	0.48	0.22*	0.16	0.03	0.13	0.09	2.12	− 0.22	1.68
eGFR	− 0.01	− 0.02	0.04	0.03	− 0.01	− 0.15	0.97	0.47	− 0.07	0.07	− 0.09	0.04

*BMI* body mass index, *SBP* systolic blood pressure, *Scr* serum creatinine, *UA* uric acid, *TCH* total cholesterol, *TG* triglycerides, *HDL-cholesterol* serum high density lipoprotein cholesterol, *LDL-cholesterol* low density lipoprotein cholesterol, *eGFR* estimated glomerular filtration ratio, *L-RSFV* left renal sinus fat volume, *R-RSFV* right renal sinus fat volume, *CI* confidence interval, *SE* standard error, *β(Beta)* standardized beta coefficient

**p < 0.01; *p < 0.05

## Discussion

There were four principal findings in this study. First, bilateral RSFV was higher in the nephrolithiasis group than in the control group. Second, bilateral nephrolithiasis patients had higher age, BMI, and SBP; higher serum levels of Scr, UA, TCH, and TG and lower HDL-cholesterol level and eGFR than healthy subjects. Third, RSFV significantly correlated with age, BMI, SBP, and eGFR in bilateral nephrolithiasis patients. Lastly, age, BMI, and LDL cholesterol were found to be independent predictors of left RSFV, and only BMI was an independent predictor of right RSFV.

Multiple imaging modalities can be used to assess the development of renal lesions in humans, including renal sinus fat accumulation, such as ultrasonography, CT, and magnetic resonance imaging [22–24]. Further, CT has been used to develop a feasible and reproducible measurement protocol for characterizing the distribution of RS fat in a sample from the Framingham MDCT cohort [20]. We delineated the RS area on axial CT images slice-by-slice and obtained the total volume of RS fat semi-automatically. The RSFV measurements theoretically might be affected by an operator-associated bias; however, we noted high intra-class and inter-class correlations, thereby ensuring the reproducibility of the results.

Our study confirms conclusions from many previous studies. Caglar et al. [25] demonstrated that RSFV and age were positively correlated for both kidneys in normal subjects, although this correlation appeared to decline after 70 years of age. Similarly, in this study, left RSFV was positively correlated with age in bilateral nephrolithiasis patients. This may allow clinicians to estimate the age-related RS fat volume changes better and help decision making. In addition, consistent with previous studies [26, 27], the right RSFV was significantly lower than left RSFV in normal subjects. This asymmetry predisposes the left RS to higher RS adipose tissue deposition. We speculate that, first, asymmetrical RS adipose tissue accumulation could be a result of the anatomical differences between the left and right renal veins. The right renal vein receives blood from only the right kidney, whereas the left renal vein receives blood from the left gonadal and adrenal veins in addition to the vein from the left kidney. The mean left renal blood flow was on average significantly lower than the right mean renal blood flow. Second, there was a wide variation in calyceal orientation, as the calyceal angles in the right and left kidneys differ. We hypothesized that a significantly higher RS adipose tissue deposition in the left kidney could possibly be an inborn structural phenomenon that might cause asymmetrical renal blood flow.

In this study, RSFV was associated with higher SBP and lower eGFR. These associations disappeared in the multivariate linear regression analysis. However, we noted a significantly higher SBP and lower eGFR in the nephrolithiasis group supporting previously published findings demonstrating that people with high BP have an increased risk of developing kidney stones, and those with kidney stones have an increased risk of developing high BP [28]. In addition, Wagner et al. [18] concluded that perivascular adipose tissue in the RS may play a role in the pathogenesis of microalbuminuria; although preliminary, our results are somewhat suggestive of this notion.

We showed that RS fat accumulation was associated with BMI in the multivariate linear regression analysis suggesting that RSFV may have an independent association with BMI. RS fat is considerably affected by visceral adipose tissue volume and has characteristics of PVAT [13]. Our results thus provide further insight regarding the pathophysiologic role of adiposity in the kidney. Dwyer et al. [29] reported that in obese rabbits, the RS mass was 61%, and obesity alone could cause renal lipomatosis. RS adipose tissue dysfunction, as is typical in obese individuals, could represent the primary mechanism involving mechanical and paracrine effects [30].

It is noteworthy that our multivariate analysis showed a close association between left RSFV and LDL-cholesterol, suggesting a possible connection between RS fat accumulation and VC. On the one hand, compression of structures within the RS, including the renal medulla, renal vein, and lymph vessels, increases renal hydro-static pressure and stimulates the renin–angiotensin–aldosterone system (RAAS) [14, 31, 32]. Activation of the RAAS promotes hypertension, atherosclerosis, and other adverse physiological effects. Conversely, as an active endocrine organ, the excessive adipose tissue in the RS may be associated with the overproduction of metabolically active substances leading to pro-inflammatory processes that mediate the association with cardiometabolic risk factors.

The present study has several limitations. First, the results were associative and observational. Therefore, we cannot establish a causal relationship between RS fat and nephrolithiasis. Second, the results may have been affected by unrecognized potentially confounding variables. To account for this possibility, we performed multivariable regression analyses that accounted for factors known to influence RSFV. Third, our study population primarily included Chinese individuals. Larger studies are needed to determine if the results of this study are generalizable to individuals of other races and ethnicity. Finally, kidney stone composition was not analyzed in this study. The mechanism underlying kidney stone formation may differ according to the stone composition and affect biochemical indices.

In conclusion, our results demonstrate the presence of RS adipose tissue accumulation in bilateral nephrolithiasis patients. Further, left RS fat was found to be correlated with age, BMI, and LDL-cholesterol in these patients. Our findings may serve as a basis for future prospective trials exploring the potential benefit of therapeutically targeting kidney stones and the cardiovascular system in nephrolithiasis patients as part of the routine management to mitigate nephrolithiasis-associated morbidity and recurrence.

## References

[1] Pfau A, Knauf F (2016). Update on nephrolithiasis: core curriculum 2016. Am J Kidney Dis.

[2] Khan SR, Canales BK (2015). Unified theory on the pathogenesis of Randall’s plaques and plugs. Urolithiasis.

[3] Khan SR (2012). Is oxidative stress, a link between nephrolithiasis and obesity, hypertension, diabetes, chronic kidney disease, metabolic syndrome?. Urol Res.

[4] Ramaswamy K, Shah O (2014). Metabolic syndrome and nephrolithiasis. Transl Androl Urol.

[5] Shavit L, Girfoglio D, Vijay V, Goldsmith D, Ferraro PM, Moochhala SH, Unwin R (2015). Vascular calcification and bone mineral density in recurrent kidney stone formers. Clin J Am Soc Nephro.

[6] Lee JJ, Pedley A, Hoffmann U, Massaro JM, Levy D, Long MT (2018). Visceral and intrahepatic fat are associated with cardiometabolic risk factors above other ectopic fat depots: the Framingham Heart Study. Am J Med.

[7] Hatem SN, Sanders P (2014). Epicardial adipose tissue and atrial fibrillation. Cardiovasc Res.

[8] van Woerden G, Gorter TM, Westenbrink BD, Willems TP, van Veldhuisen DJ, Rienstra M (2018). Epicardial fat in heart failure patients with mid-range and preserved ejection fraction. Eur J Heart Fail.

[9] Evin M, Broadhouse KM, Callaghan FM, McGrath RT, Glastras S, Kozor R, Hocking SL, Lamy J, Redheuil A, Kachenoura N, Fulcher GR, Figtree GA, Grieve SM (2016). Impact of obesity and epicardial fat on early left atrial dysfunction assessed by cardiac MRI strain analysis. Cardiovasc Diabetol.

[10] Manka D, Chatterjee TK, Stoll LL, Basford JE, Konaniah ES, Srinivasan R, Bogdanov VY, Tang Y, Blomkalns AL, Hui DY, Weintraub NL (2014). Transplanted perivascular adipose tissue accelerates injury-induced neointimal hyperplasia. Arterioscler Thromb Vasc Biol.

[11] Alkhalil M, Edmond E, Edgar L, Digby JE, Omar O, Robson MD, Choudhury RP (2018). The relationship of perivascular adipose tissue and atherosclerosis in the aorta and carotid arteries, determined by magnetic resonance imaging. Diab Vasc Dis Res.

[12] Gollasch M, Welsh DG, Schubert R (2018). Perivascular adipose tissue and the dynamic regulation of K_v_ 7 and K_ir_ channels: implications for resistant hypertension. Microcirculation.

[13] Stefan N, Häring HU, Hu FB, Schulze MB (2013). Metabolically healthy obesity: epidemiology, mechanisms, and clinical implications. Lancet Diabetes Endocrinol.

[14] Montani JP, Carroll JF, Dwyer TM, Antic V, Yang Z, Dulloo AG (2004). Ectopic fat storage in heart, blood vessels and kidneys in the pathogenesis of cardiovascular diseases. Int J Obes Relat Metab Disord.

[15] Ferrara D, Montecucco F, Dallegri F, Carbone F (2019). Impact of different ectopic fat depots on cardiovascular and metabolic diseases. J Cell Physiol.

[16] Chughtai HL, Morgan TM, Rocco M, Stacey B, Brinkley TE, Ding J, Nicklas B, Hamilton C, Hundley WG (2010). Renal sinus fat and poor blood pressure control in middle-aged and elderly individuals at risk for cardiovascular events. Hypertension.

[17] Foster MC, Hwang SJ, Porter SA, Massaro JM, Hoffmann U, Fox CS (2011). Fatty kidney, hypertension, and chronic kidney disease. Hypertension.

[18] Wagner R, Machann J, Lehmann R, Rittig K, Schick F, Lenhart J, Artunc F, Linder K, Claussen CD, Schleicher E, Fritsche A, Häring HU, Weyrich P (2012). Exercise-induced albuminuria is associated with perivascular renal sinus fat in individuals at increased risk of type 2 diabetes. Diabetologia.

[19] Murakami Y, Nagatani Y, Takahashi M, Ikeda M, Miyazawa I, Morino K, Ohkubo T, Maegawa H, Nitta N, Sakai H, Nota H, Ushio N, Murata K (2016). Renal sinus fat volume on computed tomography in middle-aged patients at risk for cardiovascular disease and its association with coronary artery calcification. Atherosclerosis.

[20] Foster MC, Hwang SJ, Porter SA, Massaro JM, Hoffmann U, Fox CS (2011). Development and reproducibility of a computed tomography-based measurement of renal sinus fat. BMC Nephrol.

[21] Levey AS, Coresh J, Greene T, Stevens LA, Zhang YL, Hendriksen S, Kusek JW, Van Lente F, Collaboration CKDE (2006). Using standardized serum creatinine values in the modification of diet in renal disease study equation for estimating glomerular filtration rate. Ann Intern Med.

[22] Nikolaidis P, Gabriel H, Khong K, Brusco M, Hammond N, Yagmai V, Casalino D, Hoff F, Patel S, Miller F (2008). Computed tomography and magnetic resonance imaging features of lesions of the renal medulla and sinus. Curr Probl Diagn Radiol.

[23] Rha SE, Byun JY, Jung SE, Oh SN, Choi Y, Lee A, Lee JM (2004). The renal sinus: pathologic spectrum and multimodality imaging approach. Radiographics.

[24] Liu BX, Sun W, Kong XQ (2019). Perirenal fat: a unique fat pad and potential target for cardiovascular disease. Angiology.

[25] Caglar V, Songur A, Acar M, Uygur R, Alkoc OA, Acar T (2014). Volumetric evaluation of fat in the renal sinus in normal subjects using stereological method on computed tomography images and its relationship with body composition. Folia Morphol.

[26] Cohen EI, Kelly SA, Edye M, Mitty HA, Bromberg JS (2009). MRI estimation of total renal volume demonstrates significant association with healthy donor weight. Eur J Radiol.

[27] Krievina G, Tretjakovs P, Skuja I, Silina V, Keisa L, Krievina D, Bahs G (2016). Ectopic adipose tissue storage in the left and the right renal sinus is asymmetric and associated with serum kidney injury molecule-1 and fibroblast growth factor-21 levels increase. EBioMedicine.

[28] Borghi L, Meschi T, Guerra A, Briganti A, Schianchi T, Allegri F, Novarini A (1999). Essential arterial hypertension and stone disease. Kidney Int.

[29] Dwyer TM, Mizelle HL, Cockrell K, Buhner P (1995). Renal sinus lipomatosis and body composition in hypertensive, obese rabbits. Int J Obes Relat Metab Disord.

[30] Shavit L, Girfoglio D, Vijay V, Goldsmith D, Ferraro PM, Moochhala SH, Unwin R (2015). Vascular calcification and bone mineral density in recurrent kidney stone formers. Clin J Am Soc Nephrol.

[31] Ott CE, Navar LG, Guyton AC (1971). Pressures in static and dynamic states from capsules implanted in the kidney. Am J Physiol.

[32] Manrique C, Lastra G, Gardner M, Sowers JR (2009). The renin angiotensin aldosterone system in hypertension: roles of insulin resistance and oxidative stress. Med Clin North Am.

